# Game-Theoretical Modeling of Interviral Conflicts Mediated by Mini-CRISPR Arrays

**DOI:** 10.3389/fmicb.2020.00381

**Published:** 2020-03-20

**Authors:** Jaime Iranzo, Guilhem Faure, Yuri I. Wolf, Eugene V. Koonin

**Affiliations:** ^1^Centro de Biotecnología y Genómica de Plantas, Universidad Politécnica de Madrid (UPM) – Instituto Nacional de Investigación y Tecnología Agraria y Alimentaria (INIA), Madrid, Spain; ^2^Institute for Biocomputation and Physics of Complex Systems (BIFI), University of Zaragoza, Zaragoza, Spain; ^3^National Center for Biotechnology Information, National Library of Medicine, National Institutes of Health, Bethesda, MD, United States; ^4^Broad Institute of MIT and Harvard, Cambridge, MA, United States

**Keywords:** CRISPR-Cas, mini-CRISPR arrays, intervirus conflicts, superinfection exclusion, game theory

## Abstract

All cellular organisms coevolve with multiple viruses, so that both virus-host and intervirus conflicts are major factors of evolution. Accordingly, hosts evolve multiple, elaborate defense systems and viruses respond by evolving means of antidefense. Although less thoroughly characterized, several dedicated mechanisms of intervirus competition have been described as well. Recently, the genomes of some bacterial and archaeal viruses have been shown to harbor CRISPR mini-arrays that typically contain a single spacer targeting a closely related virus. The involvement of mini-arrays in an intervirus conflict has been experimentally demonstrated for a pair of archaeal viruses. We model the evolution of virus-encoded CRISPR mini-arrays using a game theoretical approach. Analysis of the model reveals multiple equilibria that include mutual targeting, unidirectional targeting, no targeting, cyclic polymorphism, and bistability. The choice between these evolutionary regimes depends on the model parameters including the coinfection frequency, differential productivity of the conflicting viruses, and the fitness cost of mini-arrays. At high coinfection frequencies, the model becomes a version of the Prisoner’s dilemma in which defection, i.e., mutual targeting between the competing viruses, is the winning strategy.

## Introduction

Most microbial communities are associated with highly diverse and abundant viral populations (Perez Sepulveda et al., 2016; Duerkop, 2018; Pratama and van Elsas, 2018; Shkoporov and Hill, 2019). Although the ratio of virus particle counts to the counts of microbial cells varies within a broad range, in many habitats viruses outnumber cells by one to two orders of magnitude (Suttle, 2007; Rohwer et al., 2009; Wigington et al., 2016). A major consequence of the imbalance in viral and host abundances is that viruses of bacteria and archaea often have to compete for a limited number of host cells. To minimize the loss of productivity that occurs when two or more viruses have to share the limited resources of the same host cell, prokaryotic viruses have evolved mechanisms to prevent superinfection and cope with coinfecting viruses (Delbruck, 1945; Wagner, 1960; Nowak and May, 1994; Refardt, 2011). Among such mechanisms, those leading to superinfection exclusion in lysogenic viruses are the best studied ones, both experimentally and theoretically, from a population dynamics perspective (Lu and Henning, 1994; Sun et al., 2006). Other strategies involve increasing virulence (Eshelman et al., 2010; Refardt, 2011), sequestering diffusible intracellular products (Turner and Chao, 1998, 2003), and inducing host defense systems, such as CRISPR-Cas, against the competing virus (Erdmann et al., 2014).

Analysis of viral genomes from databases and environmental samples has revealed that viruses can co-opt parts of the CRISPR-Cas antiviral defense systems from the host and use them as weapons against competing viruses. Specifically, some viruses and prophages contain mini-CRISPR arrays with 1 or 2 spacers that target sequences from related viruses, typically infecting the same host and sometimes isolated from the same environment (Faure et al., 2019). Viruses carrying mini-arrays lack *cas* genes. However, repeats in mini-CRISPR arrays are identical to those found in the host’s CRISPR-Cas locus, suggesting compatibility of the viral mini-array with CRISPR-Cas components of the host. In some cases, pairs of viruses infecting the same host target each other through their mini-CRISPR arrays. Such mutual targeting supports the role of mini-CRISPR arrays as weapons in interviral conflicts which, combined with the *cas* genes from the host, target and destroy other viruses that could coinfect the same host. In the case of proviruses and other non-lytic viruses, mini-CRISPR arrays would constitute heterotypic superinfection exclusion mechanisms that promote long-term survival of the carrier by protecting the host from potentially lethal infections. Recently, intervirus competition among archaeal viruses carrying mini-arrays with cross-targeting spacers has been validated experimentally (Medvedeva et al., 2019).

We were interested in investigating the role of mini-arrays in interviral conflicts from an evolutionary cost vs. benefit perspective. To this end, here we develop a game-theoretical model of interviral competition mediated by mini-CRISPR arrays. The main goal of this work is to identify the conditions under which such virus-against-virus targeting becomes a successful strategy. Our results shed light on the ecological and evolutionary trade-offs that lead to the engagement of bacterial and archaeal viruses in CRISPR-mediated arms races. We provide testable predictions on the differential prevalence of mini-CRISPR arrays among viruses with different lifestyles.

## Results

### Modeling CRISPR-Mediated Competition Among Bacterial and Archaeal Viruses

We present a minimal model of interviral conflict mediated by mini-CRISPR arrays. Because interviral conflicts are driven by mixed coinfection of hosts, the coinfection probability is the main ingredient of our model. Below, we introduce a parameterization of the coinfection probability based on Bayes’ theorem and discuss it in detail. Then, we describe the mathematical model, which combines elements from game theory and ordinary differential equations, and obtain its steady state solutions for a general case. We apply the model to three scenarios that involve, respectively, two lytic viruses; a lytic virus and a temperate virus (or a provirus); and two temperate viruses or proviruses. Finally, we briefly discuss some extensions of the model to account for the loss of CRISPR spacers and the competition among more than two viruses.

#### Coinfection Probability

Let us consider two viruses,*A* and *B*, both infecting the same host. Mixed coinfections, in which viruses *A* and *B* infect the same cell, are a central ingredient of the model. From the point of view of virus *A*, the coinfection probability *p*_*B—A*_ is the probability that virus *A* encounters virus *B* during the infection process. Similarly, we define the coinfection probability for virus *B*, *p*_*A—B*_, as the probability that virus *B* encounters *A* in the course of a single infection. From the perspective of the host population, the *virus-centric* coinfection probability *p*_*B—A*_ can be understood as the conditional probability of finding a host infected by virus *B* given that the host is also infected by virus *A*. Applying Bayes’ theorem, we get

$$p_{B|A}=\frac{p_{B}}{p_{A}}\,p_{A|B}$$

where *p*_*A*_ and *p*_*B*_ are the probabilities of finding hosts infected by virus *A* and virus *B*, respectively. It is clear from this equation that the coinfection probabilities *p*_*B—A*_ and *p*_*A—B*_ can take different values. Such differences are caused by differences in abundance, infectivity, spatial distribution, host range, and any other factor that introduces an imbalance in the prevalence of viral infections. All other factors being equal, the coinfection probability will be greater for the virus with the lower abundance, the more restricted distribution, or the narrower host range.

In the case of viruses that target each other via mini-CRISPR arrays, the outcome of a coinfection event likely depends on which virus infected the host first. To account for that, we split each coinfection probability into two components, that is, *p*_*B*|*A*_ = *p*_*AB*|*A*_ + *p*_*BA*|*A*_ and *p*_*A*|*B*_ = *p*_*AB*|*B*_ + *p*_*BA*|*B*_, where the subindices *AB* and *BA* indicate the sequence in which infections occurred. We introduce the parameters *q*_*A*_ and *q*_*B*_ to denote the fractions of the coinfection events in which viruses *A* and *B* were, respectively, the first to arrive. From these definitions, it follows that *p*_*AB*|*A*_ = *q*_*A*_×*p*_*B*|*A*_ and *p*_*BA*|*B*_ = *q*_*B*_×*p*_*A*|*B*_.

#### Mathematical Model

We model the competition among two groups of viruses (species or strains), each of which can possess or lack mini-CRISPR arrays against the other. For simplicity, and without loss of generality, we normalize the productivity of an infection by a single virus that lacks mini-CRISPR arrays to make it equal to 1 (this is an arbitrary choice because multiplying all productivities by the same factor does not affect the relative composition of the population). When two viruses coinfect the same host, the offspring of each virus depends on whether they possess or lack mini-CRISPR arrays targeting each other:

(a) In the absence of such arrays, the average productivity of each virus in a mixed coinfection is given by the parameter *w* (*w*_*A*_ for virus *A*, *w*_*B*_ for virus *B*). If coinfection does not affect the total yield and both viruses are equally competitive, we can simplify *w*_*A*_ = *w*_*B*_ = 1/2. By using different values of *w*_*A*_ and *w*_*B*_, the model can account for unequal competitive abilities (*w*_*A*_≠*w*_*B*_), interference (*w*_*A*_ + *w*_*B*_ < 1) and facilitation (*w*_*A*_ + *w*_*B*_ > 1) between viruses.
(b) If only one virus has a mini-CRISPR array with a spacer against the other virus, the productivities are *1-c* for the virus that carries the array and *0* for the targeted virus. Parameter *c* (denoted as *c*_*A*_ for virus *A* and *c*_*B*_ for virus *B*) is the cost of having a mini-CRISPR array with a spacer that matches a sequence that is often closely similar to a sequence in the genome of the mini-array-carrying virus itself (although not identical because, in such a case, self-targeting would inactivate the virus’ own genome; see Discussion).
(c) If both viruses target each other, we assume that “the first that arrives wins.” This is biologically plausible because, whereas transcription and processing of mini-CRISPR arrays require some time, scanning and cleavage of the targeted viral DNA is very fast (Singh et al., 2016; Shibata et al., 2017; Raper et al., 2018; Vink et al., 2019). Therefore, unless both viruses enter the cell shortly after each other, the second virus will be incapacitated before its mini-CRISPR array can be transcribed. Mathematically, the average productivity of coinfecting viruses that target each other is *q*(1−*c*). In general, *q* and *c* are different for viruses *A* (*q*_*A*_, *c*_*A*_) and *B* (*q*_*B*_, *c*_*B*_). As explained above, *q*_*A*_ can be interpreted as the probability that virus *A* arrives first and virus *B* arrives later (and the reverse for *q*_*B*_). Note, however, that the “first who arrives wins” assumption can be easily relaxed to allow for some degree of mutual destruction upon coinfection, in which case *q*_*A*_ + *q*_*B*_ < 1.

The parameters of the model are summarized in Table 1. Competition between viruses *A* and *B* can be modeled as an evolutionary game, with the strategies being having or not having a spacer against the other virus. The fitness values of each virus are given in Table 2, under the assumption that populations of viruses *A* and *B* are homogeneous with respect to their strategies (i.e., all members either possess or lack a spacer against the other virus).

**TABLE 1:** Variables and parameters of the model.

**Symbol**	**Description**
*p*_*B—A*_	Coinfection probability for virus *A*
*p*_*A—B*_	Coinfection probability for virus *B*
*c*,*c*_*A*_,*c*_*B*_	Cost of the mini-CRISPR array
*w*,*w*_*A*_,*w*_*B*_	Viral productivity in a mixed coinfection
*q*,*q*_*A*_,*q*_*B*_	Probability that the virus survives when there is mutual targeting
*x*_*A*_,*x*_*B*_	Fraction of the viral population that harbors a mini-CRISPR array
*P*^∗^,*P*^∗∗^	Critical coinfection probabilities for the maintenance of a mini-CRISPR array

*The cost of the mini-CRISPR array and the viral productivity in a mixed coinfection are defined in relation with the basal productivity in a pure infection.*

**TABLE 2:** Mean productivity of a virus (*A*), which encounters a second virus (*B*) with probability *p*_*B—A*_, depending on the presence or absence of mutually targeting CRISPR arrays.

		**Anti-*A* spacer in virus *B***	
		**+**	–
Anti-*B* spacer in virus *A*:	+	(1-*c*_*A*_)(1-*p*_*B*|*A*_ + *q*_*A*_*p*_*B*|*A*_)	1-*c*_*A*_
	–	1-*p*_*B*|*A*_	1−(1−*w*_*A*_)*p*_*B*|*A*_

Before proceeding with the formal analysis of the system, some immediate insight can be gained from inspecting the fitness matrix of the evolutionary game. If we consider a scenario in which virus *B* lacks anti-*A* spacers, the fitness of virus *A* is given by the second column of Table 2. Accordingly, virus *A* benefits from anti-*B* spacers if *p*_*B*|*A*_ > *c*_*A*_/(1−*w*_*A*_). Conversely, if the entire population of virus *B* carries anti-*A* spacers (first column of Table 1), virus *A* will benefit from anti-*B* spacers if *p*_*B*|*A*_ > *c*_*A*_/(*q*_*A*_−*q*_*A*_*c*_*A*_ + *c*_*A*_). Thus, the outcome of the evolutionary process is determined by a trade-off between the cost of the CRISPR mini-array and the potential benefit conferred by the spacer against the competing virus during coinfections.

To analyze the general case of mixed populations, let us introduce new variables *x*_*A*_ and *x*_*B*_, both with values between *0* and *1*, which represent the fraction of viruses of each strain that harbor CRISPR spacers against the other strain. Conversely, 1−*x*_*A*_ and 1−*x*_*B*_ are the relative sizes of the spacer-free subpopulations. Focusing on strain *A*, the fitness of viruses with and without anti-*B* spacers (denoted as *f*_*A,1*_ and *f*_*A,2*_, respectively) results from multiplying the fitness matrix in Table 2 by the fractions of viruses in strain *B* that harbor and lack anti-*A* spacers:

$$f_{A,1}\,\left(x_{B}\right)=\left(1-c_{A}\right)\,\left(1-p_{B|A}+q_{A}\,p_{B|A}\right)\,x_{B}+$$

(1) $$\left(1-c_{A}\right)\,\left(1-x_{B}\right)$$

$$f_{A,2}\,\left(x_{B}\right)=\left(1-p_{B|A}\right)\,x_{B}+\left(1-\left(1-w_{A}\right)\,p_{B|A}\right)\,\left(1-x_{B}\right)$$

Because of the complexity of virus-host interaction networks in natural microbial communities, it is unlikely that the host ranges or geographical distributions of two viruses completely overlap (Flores et al., 2013; Munson-McGee et al., 2018). The existence of strain-exclusive reservoirs partially decouples the population dynamics of different viruses, such that the coinfection probabilities cannot be easily calculated by simply considering both viruses and their shared host. Moreover, even in simple models with one virus and one host, the addition of realistic features, such as heterogeneous spatial structure or antiviral defense systems, dramatically alters the infection dynamics, stabilizing virus and hosts populations toward an equilibrium state (Heilmann et al., 2012; Iranzo et al., 2015). Because of these reasons, we do not attempt to model the coinfection dynamics and, instead, treat the coinfection probabilities as independent parameters, under the assumption that these are governed, to a large extent, by the overlap between the ecological niches of the competing viruses. By adopting the framework of evolutionary game theory, we can study the fraction of viruses with and without spacers for each strain without explicitly modeling the host population, the absolute sizes of viral populations, and the possible differences in the infection rates of each virus. Thus, the dynamics of the mixed population can be represented by the following replicator equations:

(2) $$\left\{ \begin{array}{cc} {\dot{x}}_{A} & =x_{A}\,\left(f_{A,1}\,\left(x_{B}\right)-\phi_{A}\,\left(x_{A},x_{B}\right)\right)\\ {\dot{x}}_{B} & =x_{B}\,\left(f_{B,1}\,\left(x_{A}\right)-\phi_{B}\,\left(x_{A},x_{B}\right)\right) \end{array}\right.$$

where ϕ_*A*_ is the mean fitness of strain *A*, calculated as

(3) $$\phi_{A}=x_{A}\,f_{A,1}\,\left(x_{B}\right)+\left(1-x_{A}\right)\,f_{A,2}\,\left(x_{B}\right)$$

The mean fitness (ϕ_*B*_) and class–specific fitness values (*f*_*B,1*_ and *f*_*B,2*_) for strain *B* are obtained by exchanging subindices *A* and *B* in Eqs 3 and 1, respectively. After some manipulation, the equations that describe the population dynamics can be rewritten in a more informative form as

(4) $$\left\{ \begin{array}{cc} {\dot{x}}_{A} & =x_{A}\,\left(1-x_{A}\right)\,\left(a+b\,x_{B}\right)\\ {\dot{x}}_{B} & =x_{B}\,\left(1-x_{B}\right)\,\left(a^{\prime }+b^{\prime }\,x_{A}\right) \end{array}\right.$$

where

(5) $$a=\left(1-w_{A}\right)\,p_{B|A}-c_{A}$$

(6) $$b=p_{B|A}\,\left(q_{A}-q_{A}\,c_{A}+c_{A}+w_{A}-1\right)$$

(7) $$a^{\prime }=\left(1-w_{B}\right)\,p_{A|B}-c_{B}$$

(8) $$b^{\prime }=p_{A|B}\,\left(q_{B}-q_{B}\,c_{B}+c_{B}+w_{B}-1\right)$$

#### General Solution of the Model

**FIGURE 1:**
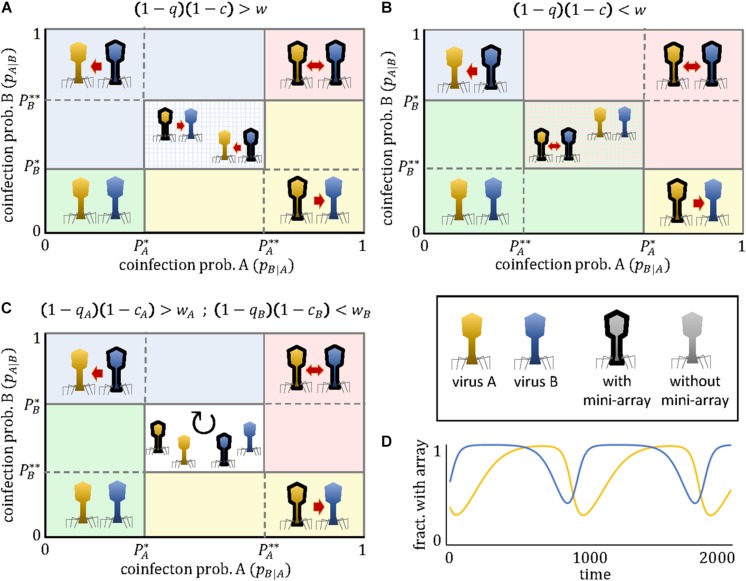
Outcomes of the mini-CRISPR array-mediated virus arms race as a function of the coinfection probabilities experienced by each virus. Depending on the relative ordering of the critical thresholds *P*^∗^ and *P*^∗∗^, three qualitatively different cases exist **(A–C)**. The presence of a mini-CRISPR array in a virus is denoted by black borders and a directed arrow pointing to the other virus. Mutual targeting is indicated by bidirectional arrows. In panels **(A)** and **(B)** the central region correspond to a bistable state in which two outcomes are possible. The central region in panel **(C)** corresponds to a state of cyclic polymorphism involving subpopulations with and without mini-CRISPR arrays. An example of such cyclic dynamics is shown in panel **(D)**. Parameter values in panel **(D)** are *c* = 0.1, *w*_*A*_ = 0.8, *w*_*B*_ = 0.5, *q*_*A*_ = 0.5, *q*_*B*_ = 0.2, *p*_*B*|*A*_ = 0.2, *p*_*A*|*B*_ = 0.3.

A comprehensive analysis of the solutions of Eq. 4 indicates that the outcome of the evolutionary arms race between the two viruses is given by the relative values of the coinfection probabilities *p*_*B—A*_ and *p*_*A—B*_ with respect to two pairs of critical thresholds, $P_{A}^{*}$ and $P_{A}^{**}$ for virus *A*, and $P_{B}^{*}$ and $P_{B}^{**}$ for virus *B*. The expressions for these critical thresholds are

(9) $$P^{*}=\frac{c}{1-w}$$

(10) $$P^{**}=\frac{c}{q-q\,c+c}$$

(note that we omit the subindices *A* and *B* for simplicity). An intuitive interpretation of these critical thresholds is that they represent the coinfection probabilities for which the benefit of targeting the competitor virus outweighs the cost of the CRIPSR array, either when the competitor lacks a cross-targeting spacer and hence does not engage in the arms race (*P*^∗^) or when it does (*P*^∗∗^). Depending on the coinfection rate, the evolutionary arms race can lead to 5 qualitatively different regimes (Figure 1):

(a) No targeting if $p_{B|A}<P_{A}^{*}$ and $p_{A|B}<P_{B}^{*}$
(b) Unidirectional targeting, with *A* targeting *B* if $p_{B|A}>P_{A}^{*}$ and $p_{A|B}<P_{B}^{**}$
(c) Unidirectional targeting, with *B* targeting *A* if $p_{B|A}<P_{A}^{**}$ and $p_{A|B}>P_{B}^{*}$
(d) Mutual targeting if $p_{B|A}>P_{A}^{**}$ and $p_{A|B}>P_{B}^{**}$
(e) Cyclic dominance, with an alternation of targeting and non-targeting subpopulations, if $P_{A}^{*}$< *p*_*B—A*_ <$P_{A}^{**}$ and $P_{B}^{**}$< *p*_*A—B*_ <$P_{B}^{*}$; or else, if $P_{A}^{**}$< *p*_*B—A*_ <$P_{A}^{*}$ and $P_{B}^{*}$< *p*_*A—B*_ <$P_{B}^{**}$

Two important observations are pertinent. First, with the exception of the last regime, the population always reaches an equilibrium state in which all viruses of a given type (*A* or *B*) either possess or lack the mini-CRISPR array. Second, for some parameter combinations, the equilibrium is not unique, that is, the system displays bistability. Specifically, if $P_{A}^{*}$< *p*_*B—A*_ <$P_{A}^{**}$ and $P_{B}^{*}$< *p*_*A—B*_ <$P_{B}^{**}$, unidirectional targeting, either from *A* to *B* or from *B* to *A* (but not mutual targeting), will evolve. Which of the two possible equilibria is reached in that bistable regime, depends on which virus acquired the spacer first. The existence of this regime implies that instances of unidirectional targeting can be found even if viruses *A* and *B* are identical in all their parameters. Alternatively, if $P_{A}^{**}$< *p*_*B—A*_ <$P_{A}^{*}$ and $P_{B}^{**}$< *p*_*A—B*_ <$P_{B}^{*}$, the population can evolve toward the regimes of mutual targeting or no targeting, and both outcomes are stable.

It follows from Eq. 9 that, in order to maintain a mini-CRISPR array, the probability of coinfection must be greater than the cost of the array. Moreover, possession of mini-CRISPR arrays is more advantageous for strains with low yields in mixed coinfections (low *w*_*A*_) because those would benefit the most from curtailing other, more efficient coinfecting strains.

#### Competition Between Two Lytic Viruses

When considering 2 lytic viruses, the model can be simplified by noting that any of them can be the first to enter the host cell with the same probability, that is, *q* = 1/2. Moreover, empirical data indicate that viruses that target each other are typically closely related (Faure et al., 2019; Medvedeva et al., 2019). Therefore, it seems reasonable to assume equal fitness costs (*c*_*A*_ = *c*_*B*_ = *c*). Similarly, unless one of the viruses has specialized to exploit the other (which is unlikely given the close relatedness), similar coinfection yields (*w*_*A*_ = *w*_*B*_ = *w*) can be expected. With these simplifications, the threshold coinfection probabilities required for the maintenance of mini-CRISPR arrays (Eqs 9–10) become:

(11) $$P^{*}=\frac{c}{1-w}$$

(12) $$P^{**}=\frac{2\,c}{1+c}$$

**FIGURE 2:**
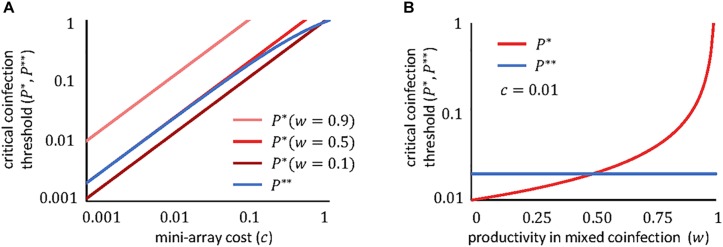
Dependency of the critical coinfection thresholds *P*^∗^ (red) and *P*^∗∗^ (blue) on the cost of the mini-CRISPR array *c* **(A)** and on the viral productivity in mixed coinfections *w* **(B)**. We fixed *q* = 1/2, which is appropriate if both viruses are equally likely to enter the cell first. If both viruses obtain even yields in mixed confections, *w* = 1/2 corresponds to the case where viruses do not interfere with each other; *w* < 1/2 if there is interference; and *w* > 1/2 if there is facilitation. Note the logarithmic scale of the axes.

The dependencies of these thresholds on the fitness cost and the productivity in mixed coinfections are plotted in Figure 2. Clearly, in most realistic scenarios, the critical coinfection probabilities have the same order of magnitude as the fitness cost (the exception would be for viruses whose productivity remains largely unaffected when they coinfect with a second virus, that is, if *w*≈1). Furthermore, the greater the degree of interference (that is, the smaller the value of *w*), the lower the critical coinfection threshold.

**FIGURE 3:**
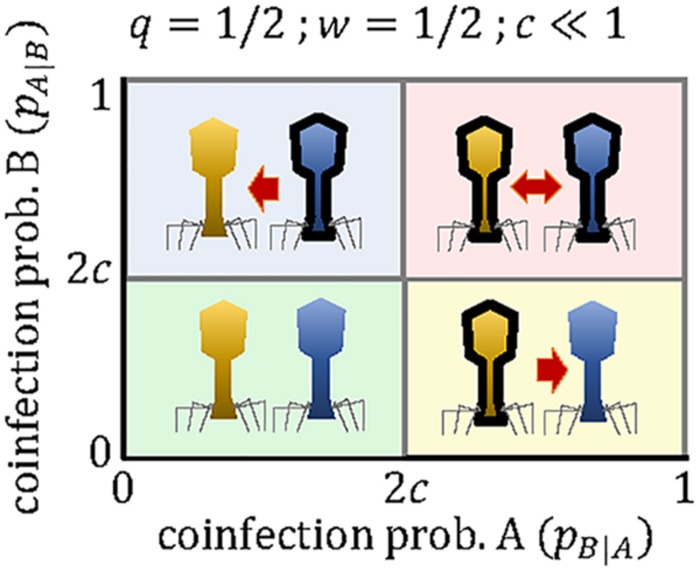
Outcomes of the mini-CRISPR array-mediated virus arms race in the case of two lytic viruses, whose productivity in mixed coinfections is half of their productivity in a pure, single infection. Each virus will maintain mini-CRISPR arrays targeting the other virus if the probability of coinfection is greater than twice the cost of the array. Viruses *A* and *B* are represented in yellow and blue, respectively. Black borders denote the presence of a mini-CRISPR array.

If both viruses can use the molecular machinery synthesized by the other virus, and in the absence of other sources of interference, the productivity of each virus in a mixed coinfection will be half of the productivity in a pure infection (that is, *w*≈1/2). If we also assume that the cost of the array is small (*c*≪1), the critical coinfection probabilities in Eqs 11–12 tend to the same value, *P*^∗∗^≈*P*^∗^ = *2c*. The possible evolutionary outcomes under this scenario become much simpler, with each virus maintaining a mini-CRISPR array against the other virus only if its coinfection probability is greater than twice the cost of the array (Figure 3). In contrast, strong interference between coinfecting viruses (*w* < 1/2) introduces additional outcomes and broadens the range of conditions in which unidirectional targeting can evolve (Figure 1A).

#### Competition Between a Lytic and a Temperate Viruses

Let us now consider a scenario with one lytic (*L*) and one temperate (*T*) virus. By temperate virus, we refer both to proviruses integrated in the host genome and to non-lytic viruses that produce non-lethal chronic infections. Although the latter are particularly common in archaea (Prangishvili et al., 2017), actively replicating temperate phages also constitute some of the most abundant viruses in the human gut virome (Shkoporov et al., 2018). Based on the different lifestyles of lytic and temperate viruses, the most likely scenario for a mixed coinfection corresponds to the superinfection of a cell that already hosts a temperate virus by an incoming lytic virus. In this scenario, the mini-CRISPR arrays carried by the temperate virus, combined with the *cas* genes from the host, act as a heterotypic superinfection exclusion mechanism that protects the host from the lytic virus. Importantly, such a mechanism remains effective even if the lytic virus harbors a mini-CRISPR array against the “resident” temperate virus. In our model, this can be captured by setting *q*_*L*_ = 0 and *q*_*T*_ = 1. Note, however, that unidirectional targeting of the temperate virus by the lytic virus leads to the elimination of the temperate virus and production of pure offspring of the lytic virus. If the temperate virus is present as a provirus, its destruction will also lead to the degradation of the host genome; in that case, we assume that replication of the lytic virus remains unaffected by host genome degradation. Because the cost of carrying a spacer can differ among viruses with distinct lifestyles, we consider virus-specific cost parameters, *c*_*L*_ and *c*_*T*_. Provirus induction is implicitly modeled by the parameter *w*_*T*_, which jointly represents the probability of induction and the fraction of viral particles that successfully insert as a provirus in a new host.

As in the previous cases, the outcome of the evolutionary process depends on the relative values of the coinfection probabilities *p*_*L—T*_ and *p*_*T—L*_ with respect to three critical thresholds:

(13) $$P_{T}^{*}=\frac{c_{T}}{1-w_{T}}$$

(14) $$P_{T}^{**}=c_{T}$$

(15) $$P_{L}^{*}=\frac{c_{L}}{1-w_{L}}$$

(there is an additional threshold for the lytic virus at $P_{L}^{**}$ =1, but it is irrelevant because, by definition, *p*_*T*|*L*_≤1). Once again, *P*^∗^ and *P*^∗∗^ represent the critical coinfection probabilities beyond which it becomes profitable to target the other virus that either does (*P*^∗∗^) or does not engage (*P*^∗^) in the arms race via its mini-CRISPR array.

**FIGURE 4:**
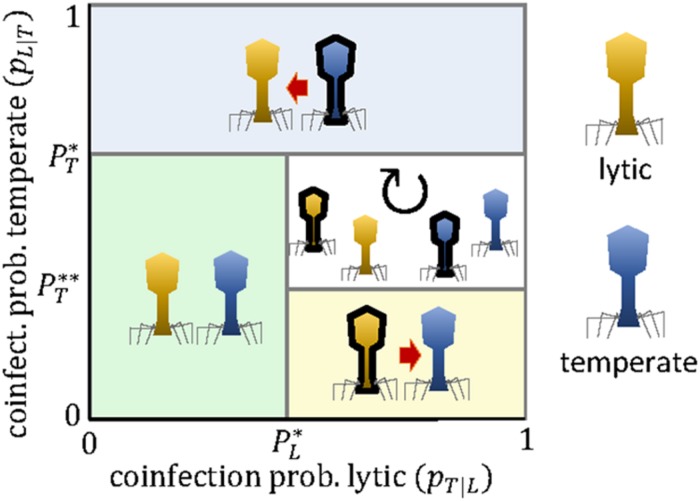
Evolutionary outcomes for the arms race between a lytic virus (*L*, yellow) and a temperate virus (*T*, blue) as a function of the coinfection probabilities *p*_*T—L*_ and *p*_*L—T*_. Black borders denote the presence of a mini-CRISPR array. The expressions for the critical coinfection thresholds $P_{L}^{*}$, $P_{T}^{*}$, and $P_{T}^{**}$ are given by Eqs 13–15.

Figure 4 shows that competition among lytic and temperate viruses can lead to unidirectional targeting or cyclic polymorphism, but not to stable, reciprocal targeting. The reason is that, once the temperate virus engages in the arms race, the lytic virus does not benefit anymore from carrying the mini-CRISPR array.

The outcome of the competition between a lytic and a temperate virus depends on (i) whether the latter is an actively replicating non-lytic virus or a provirus. For a provirus, we must distinguish (ii) whether or not it provides the host with CRISPR-independent immunity against subsequent infection by the lytic virus, and (iii) whether such immunity can be overcome by a lytic virus that carries a mini-array against the temperate virus. Let us start with the case of an actively replicating non-lytic virus. Because the long-term productivity of chronic viruses is tightly linked to the survival of the host, superinfection by lytic viruses severely reduces their fitness (*w*_*T*_≪1). In terms of the model, this implies that temperate viruses can maintain mini-arrays against lytic viruses as long as the coinfection probability is greater than the cost of the mini-array ($P_{T}^{*}$≈$P_{T}^{**}$ = *c*_*T*_). Thus, frequent encounters between the temperate and the lytic virus will result in unidirectional targeting from the former toward the latter. The same reasoning applies to those proviruses that do not provide immunity against a given lytic virus. However, many proviruses encode superinfection exclusion mechanisms that prevent infection by closely related viruses and, sometimes, even by distantly related ones (Dedrick et al., 2017; Gentile et al., 2019; Mavrich and Hatfull, 2019). Some of these mechanisms act at a very early stage of infection (e.g., inhibiting virus attachment or blocking the release of viral DNA into the cell), so that mini-CRISPR arrays would not help the incoming virus to superinfect the host. In those cases, CRISPR-mediated targeting becomes useless both for the temperate virus (which does not need it) and for the lytic virus (which does not benefit from it). This situation is not accounted for by the game matrix of the model (Table 2) but it is easy to see that it leads to no targeting among the competing viruses. Some superinfection exclusion mechanisms act at later stages of the infection process, which could allow the lytic virus to use mini-CRISPR arrays to target and destroy key proviral genes and escape immunity. This scenario equals to setting null productivity for the lytic virus upon coinfection in the absence of CRISPR-mediated targeting (*w*_*L*_ = 0, *w*_*T*_≈1), and leads to a cyclic targeting dynamics (no targeting, lytic to provirus, mutual targeting, provirus to lytic, and no targeting again) if the coinfection probabilities for both viruses are greater than the cost of the mini-array ($P_{T}^{*}$ = ∞, $P_{T}^{**}$ = *c*_*T*_, $P_{L}^{*}$ = *c*_*L*_).

#### Competition Between Two Temperate Viruses

Let us consider a scenario in which a temperate virus (*A*) infects a cell that already hosts another temperate virus or provirus (*B*). Unlike in the previous section, which dealt with viruses that qualitatively differ in their lifestyles, here, the reverse scenario in which *B* infects a cell that already hosts *A* is also possible and, once the population reaches equilibrium, equally likely. Accordingly, to apply the general model to 2 temperate viruses, we set the parameter *q* = 1/2. Additionally, to study competition among temperate viruses, it is necessary to distinguish between the cases of actively replicating non-lytic viruses and proviruses.

The model for the competition between two actively replicating temperate viruses is formally the same as for two lytic viruses, with the critical coinfection thresholds given by Eqs 11–12 (Figure 2). By contrast, when considering temperate viruses that become proviruses upon infection, unidirectional targeting of a resident provirus by an incoming virus leads to degradation of the host genome which, obviously, prevents the incoming virus from inserting into the host genome. This peculiarity of the competition between proviruses requires modifying the payoff matrix in Table 2, such that the fitness of virus *A* when it targets virus *B* becomes (1-*c*_*A*_)(1-*p*_*B*|*A*_ + *q*_*A*_*p*_*B*|*A*_), regardless of whether virus *B* targets *A* or not. With this modification, after substituting *q* = 1/2, the critical threshold *P*^∗^ for the competition of 2 proviruses becomes

(16) $$P^{*}=\frac{2\,c}{1+c-2\,w}$$

whereas *P*^∗∗^ remains the same as in Eq. 12.

For both proviruses and actively replicating non-lytic viruses, the possible evolutionary outcomes will depend on the effect of coinfection for the long-term productivity of the temperate virus. In the case of actively replicating non-lytic viruses, it seems reasonable to assume that coinfection substantially decreases viral productivity. To get an approximate idea of how that affects the evolutionary outcome, if the productivity of the resident virus decreases to half of its original value, the evolutionary regimes will coincide with those shown for lytic viruses in Figure 3. Specifically, each virus will target the other if the coinfection probability is greater than twice the cost of the mini-array. In the case of proviruses, the quantitative effect of coinfection on long-term survival is more difficult to assess. In the absence of superinfection exclusion, as long as the arrival of a second provirus does not severely affect survival of the resident provirus (*w*≥1/2), the possible evolutionary outcomes would be those shown in Figure 1B, with *P*^∗∗^≈2*c* and *P*^∗^≥1. Under this assumption, we would expect either no targeting or mutual targeting between proviruses (but not unidirectional targeting), with mutual targeting evolving only if the coinfection probability of both viruses is greater than twice the cost of the mini-array. The same outcomes are predicted if one or both proviruses possess superinfection exclusion mechanisms that can be overcome through CRISPR-mediated targeting (this scenario corresponds to setting *w* = 1/2, combining the facts that only the resident provirus can replicate and that the probability of arriving first is 1/2). In contrast, if superinfection exclusion occurs and cannot be counteracted by CRISPR-mediated targeting, the benefit of harboring the mini-array vanishes for every provirus that encodes superinfection exclusion mechanisms (if only one does, the other can still benefit from the mini-array and evolve unidirectional targeting if the coinfection probability is greater than twice the cost of the mini-array).

#### Loss or Decay of CRISPR Spacers in Mini-Arrays

In the model discussed so far, the only factor that opposes the engagement of viruses into CRISPR-mediated arms races is the fitness cost of mini-arrays. Motivated by the fact that prokaryotic genome evolution displays a consistent deletion bias (Mira et al., 2001; Kuo and Ochman, 2009) and by specific observation on the loss of CRISPR spacers (Gudbergsdottir et al., 2011; Erdmann et al., 2014; Liu et al., 2016), here we extend the model to explore the contribution of non-adaptive loss or decay of mini-array spacers to the outcome of CRISPR-mediated interviral conflicts. Although the general solution becomes more complicated, the main finding is that, for a realistic range of parameter values, fitness cost and mini-array loss rate are exchangeable. Thus, all statement made above in terms of the mini-array cost, can be reinterpreted in terms of the spontaneous loss rate of mini-arrays.

Mini-array loss can be easily incorporated into the present framework as follows. If spacers are lost at a fixed rate *d*, Eq. 2 becomes a replicator-mutator equation:

(17) $$\left\{ \begin{array}{cc} {\dot{x}}_{A} & =x_{A}\,\left(\left(1-d\right)\,f_{A,1}\,\left(x_{B}\right)-\phi_{A}\,\left(x_{A},x_{B}\right)\right)\\ {\dot{x}}_{B} & =x_{B}\,\left(\left(1-d\right)\,f_{B,1}\,\left(x_{A}\right)-\phi_{B}\,\left(x_{A},x_{B}\right)\right) \end{array}\right.$$

with the same fitness parameters *f*_*A,1*_, *f*_*A,2*_, *f*_*B,1*_, *f*_*B,2*_, ϕ_*A*_, ϕ_*B*_ as before. For simplicity, we present the equations for *w* = 1/2 and omit subindices unless necessary to prevent ambiguity. The first critical coinfection threshold becomes

(18) $$P_{A}^{*}=P_{B}^{*}=2\,\left(c+d-c\,d\right)$$

The general expression for the second critical threshold becomes more complicated because it indirectly depends, for a given virus, on the coinfection probability for the other virus. Specifically,

(19) $$P_{A}^{**}=\frac{2\,\left(c+d-c\,d\right)}{1+\left(1-d/s_{B}\right)\,\left(1-2\,\left(1-c\right)\,\left(1-q\right)\,\left(1-d\right)\right)}$$

where

(20) $$s_{B}=\frac{p_{A|B}/2-c}{1-c}$$

In these expressions, *s*_*B*_ is the relative reduction in the fitness of virus *B* associated with the loss of the CRISPR mini-array (i.e., the selection coefficient against CRISPR loss), provided that virus *A* does not engage in the arms race. Similar expressions are obtained for $P_{B}^{**}$ and *s*_*A*_ by replacing subindices *A* and *B*. Due to their dependency on *p*_*A—B*_ and *p*_*B—A*_, the critical coinfection thresholds $P_{A}^{**}$ and $P_{B}^{**}$ are no longer straight lines (as represented in Figures 1, 3, 4) but curves in the *p*_*A—B*_ vs. *p*_*B—A*_ parameter space. Nevertheless, the parameter combinations that lead to no targeting, unidirectional targeting, and mutual targeting can still be obtained by applying the rules from Figure 1. Moreover, if the loss rate is small compared to the strength of selection (*d*≪*s*_*A*_, *d*≪*s*_*B*_), Figure 1 also provides an approximate description of the bistable regimes (in that case, Figure 1A applies if (1−*q*)(1−*c*)(1−*d*) > 1/2 and Figure 1B if (1−*q*)(1−*c*)(1−*d*) < 1/2). For larger values of the loss rate, the bistable regimes are determined by a more convoluted set of equations (not shown here), and the range of parameters that allow for bistability shrinks as the loss rate grows.

The expressions from Eqs 18–20 fully apply to a loss-driven scenario where the mini-CRISPR array entails no cost (*c=0*), and spacer loss is the only force counteracting selection for the mini-CRISPR array. To further simplify the final expressions, we assume that the loss rate is small compared to the strength of selection (note that, if *c=0*, such condition becomes 2*d*≪*p*_*A*|*B*_, 2*d*≪*p*_*B*|*A*_ and it is expected to hold if coinfections are frequent enough to promote the maintenance of the mini-CRISPR array). These considerations lead to the critical thresholds *P*^∗^ = *2d* and *P*^∗∗^ = *d*/(*q*−*qd* + *d*), which coincide with the expressions in the original model (Eqs 9–10) but with the cost of the array replaced by the loss rate.

To summarize, the qualitative results of the simple model are robust to the introduction of modest rates of spacer loss. An obvious quantitative difference is that, because loss of spacers continuously produces spacer-free viruses, the populations that engage in the arms race will be polymorphic. For example, in a unidirectional targeting scenario where strain *A* carries the anti-*B* spacer, only a fraction *x*_*A*_ = 1−*d*/*s*_*A*_ of virus *A* will have the spacer.

## Discussion

Competition among bacterial and archaeal viruses can lead to a mini-CRISPR array-mediated interviral arms race if coinfections are frequent enough. The model described here predicts how frequent coinfections must be so that it becomes advantageous for a virus to carry a mini-CRISPR array targeting a competitor. Precise quantification of such threshold requires measuring the values of two key parameters: (i) the cost of maintaining a mini-CRISPR array with the virus-targeting spacer, and (ii) the productivity of a virus lacking a mini-CRISPR array in a mixed coinfection relative to the productivity of that same virus in isolation. For two lytic viruses, a necessary (and often sufficient) condition for the emergence of mutual targeting is that the coinfection probability is greater than twice the cost of having the array. The same condition holds for the emergence of mutual targeting between two temperate viruses (either proviruses or actively replicating non-lytic viruses). For the competition between lytic and temperate viruses, the model predicts evolution of unidirectional targeting, from the temperate virus to the lytic virus, or cyclic targeting dynamics if the coinfection probability exceeds the cost of the mini-array. Mutual targeting between lytic and temperate viruses is unstable because lytic viruses only benefit from targeting a temperate virus if the latter does not already target the lytic virus. These results emphasize the interplay between superinfection exclusion and CRISPR-mediated targeting in interviral conflicts that involve prophages. Indeed, the outcome of the mini-CRISPR array-mediated arms race critically depends on whether the competing viruses encode mechanisms to prevent superinfection and whether such mechanisms can be vanquished by viruses that carry mini-CRISPR arrays.

Our analysis sheds light on the effect of viral interference and facilitation on interviral conflicts, and predicts that mini-CRISPR arrays are more likely to evolve in pairs of closely related viruses that strongly interfere with each other when coinfecting the same host cell. Another prediction that arises from the model is that mini-CRISPR arrays should be more frequent in “specialist” viruses (viruses with narrow host and geographical ranges) than in “generalist” ones, especially if the virus-host interaction network is nested as suggested by empirical data (Flores et al., 2013). The reason is that specialist viruses in a nested virus-host interaction network are subject to a higher frequency of coinfection and have more predictable coinfecting partners than generalist viruses. This prediction of the model will be put to test as data on virus host ranges accumulate.

Empirically observed instances of virus pairs with mutually targeting CRISPR-arrays often involve closely related viruses (based on the similarity of the terminase large subunit) with high sequence identity in their protospacer regions (Faure et al., 2019). As a result, a major contribution to the cost of the array could result from primed adaptation (Datsenko et al., 2012) induced by the spacer against its own carrier. The effects of primed adaptation on the propagation of phages and plasmids strongly depend on the model system, the spacer sequence, and the genetic background of the host (Datsenko et al., 2012; Fineran et al., 2014; Xue et al., 2015; Strotskaya et al., 2017). Overall, the available experimental evidence suggests that mobile genetic elements could incur a substantial fitness cost (on the order of 0.1 or greater) for carrying an array with imperfect self-targeting spacers (Fineran et al., 2014; Xue et al., 2015). If these observations can be extrapolated to natural conditions in hosts other than *E. coli*, they would imply that the CRISPR-mediated arms race among closely related viruses can only be supported if coinfections with a competing virus represent a substantial fraction (about 10% or greater) of the infections by a given virus, or if spacers are divergent enough from the homologous sequence in the virus’ own genome to avoid primed adaptation. An alternative possibility is that viruses that employ CRISPR arrays to target competitors possess mechanisms to inhibit primed adaptation, as has been suggested for bacteriophage T7 (Strotskaya et al., 2017). In the absence of primed adaptation, the cost of mini-CRISPR arrays is probably small. In that case, the spread of mini-arrays can still be limited by spacer loss due to recombination between the repeats. When modified to include spacer loss, the conclusions of our study remain qualitatively unchanged, in the sense that, for practical purposes, the loss rate and the cost of the mini-array are exchangeable parameters.

The model described here is simple, yet capable to produce a broad variety of evolutionary outcomes. Specifically, equilibrium states with mutual targeting, unidirectional targeting, no targeting, cyclic polymorphism, and bistability are recovered. The asymmetry that leads to unidirectional targeting most often results from different coinfection probabilities for each virus. Thus, if virus *A* encounters virus *B* more frequently than *B* encounters *A* (e.g., because virus *B* has a broader range or greater abundance), it is more likely that only *A* would carry a spacer against *B*. Other causes that could lead to asymmetric outcomes would be unequal fitness costs and different yields in coinfection (Refardt, 2011), with lower yields promoting the maintenance of CRISPR mini-arrays. Furthermore, unidirectional targeting could evolve among viruses that are functionally and ecologically equivalent (i.e., equal in all their parameters) if the cost of the CRISPR array is outweighed by the benefit of targeting an array-free, but not an array-carrying, virus.

The model can be readily generalized to include three or more competing viruses with different coinfection probabilities. In that case, the outcome of the competition depends on how the fitness cost scales with the number of targeted viruses. In particular, if the cost increases linearly, the problem can be reduced to a combination of pairwise interactions, each of which can be separately analyzed with the 2-virus model. More specifically, the equations of the 2-virus model can be simply applied to each pair of viruses to determine if they carry spacers against the other. A notable aspect of the intervirus arms race becomes manifest in the regime of frequent coinfections with mutual targeting. In this regime, viruses engage in an analog of the Prisoner’s Dilemma (Axelrod and Hamilton, 1981; Maynard Smith, 1982), where having and not having a mini-CRISPR array targeting the competing virus becomes analogous to defecting and “cooperating” (not defecting), respectively. When coinfections are frequent, the best strategy for each virus is to target the competitor (defect), which leads to the evolution of reciprocal targeting. Paradoxically, the overall fitness that both viruses obtain when they defect and target each other is lower than the fitness that they would obtain if they both pursued their mutual benefit and did not engage in an arms race. Qualitatively similar outcomes (although based on different mechanisms) have been previously observed in the experimental evolution of phages at high multiplicity of infection (Turner and Chao, 1999) or in metapopulations with unrestricted migration (Kerr et al., 2006; Eshelman et al., 2010), suggesting that evolutionary dilemmas leading to suboptimal overall fitness could be a characteristic (although not universal, see Turner and Chao, 2003) feature of interviral conflicts.

Notwithstanding the possible sampling bias, most empirical instances of viral mini-CRISPR arrays targeting other viruses involve prophages or non-lytic archaeal viruses. Because the formal conditions required for the evolution of mini-CRISPR arrays in lytic and temperate viruses are not dramatically different, we propose that the higher prevalence of mini-CRISPR arrays in temperate viruses results from a greater frequency of coinfection in these viruses. Indeed, single-cell sequencing of a hyperthermophilic archaeal community has recently shown that most archaeal cells harbor one or more viruses (Munson-McGee et al., 2018) which are, presumably, temperate. We expect that future studies quantifying the frequency of coinfection in environmental samples will contribute to clarify whether lytic and temperate viruses substantially differ in their heterotypic coinfection rates. A second factor that could help explaining the higher prevalence of mini-CRISPR arrays in temperate viruses is their closer and temporally extended association with the host, which would give temperate viruses more opportunities to integrate spacers through recombination with the host CRISPR array and/or via CRISPR adaptation. One or the other of these spacer acquisition mechanisms are likely to operate in different virus-host systems. In mini-array carrying phages, the leader sequences in front of the mini-arrays differ from those in the hosts, presumably precluding adaptation and limiting spacer acquisition to the recombinational route (Faure et al., 2019). By contrast, in archaeal viruses, the leaders are identical to those of the host suggesting that adaptation could occur directly in the virus mini-array (Medvedeva et al., 2019).

From an evolutionary perspective, our model addresses the role of selection and neutral loss in the fate of mini-CRISPR arrays. However, it does not include other sources of genotypic variation that fuel the evolutionary process, such as mini-array acquisition and mutation of target sequences. The acquisition of spacers was not included in our model because of the uncertainty about the mechanism and also because it is unclear whether spacer capture is a limiting factor for the observed distribution of mini-CRISPR arrays in viral taxa. This aspect will need to be revisited as more empirical evidence becomes available. Another relevant process that is not covered in this work is the evolutionary escape from CRISPR targeting through mutations in viral proto-spacers. Because the long-term efficacy of proto-spacer mutation as an escape strategy depends on the rate at which new spacers are acquired, the design of more realistic coevolutionary models will also require a better understanding of the dynamics of spacer and mini-array acquisition in viral populations. Further refinements to the model should also include an explicit consideration of the host and virus population dynamics and how changes in abundance reflect on coinfection probabilities. Such an explicit modeling of time-dependent coinfection dynamics, which is not part of the present model, can shed light on whether the larger fluctuations of host populations associated with the proliferation of lytic viruses lead to fundamental differences in the fate and efficiency of mini-CRISPR arrays, as predicted for “regular,” cell-borne arrays (Iranzo et al., 2013).

There could be additional potential benefits that mini-CRISPR arrays might provide to the carrier virus and that are independent of interviral conflict but rather have to do with overcoming the host defenses. For example, high levels of transcription of the mini-CRISPR array could potentially lead to competitive exclusion of the host spacers from the CRISPR-Cas effector modules, protecting the virus from targeting. Additionally, repeats in the mini-CRISPR array could promote the insertion of the virus within the host CRISPR array, thus impairing the host immune response; indeed, insertion of proviruses into CRISPR arrays has been observed in several bacterial genomes (Faure et al., 2019; Varble et al., 2019). These scenarios could be immediately incorporated into the present framework as a negative-signed contribution to the fitness cost of the mini-array (parameter *c* in the model). Nevertheless, the fact that the spacers in CRISPR mini-arrays typically target closely related viruses and the observed instances of mutual targeting strongly suggest that the main role of mini-CRISPR arrays is related to interviral conflict.

Finally, it is worth noting that the modeling framework developed here can be generalized beyond viruses, to plasmids that might carry mini-arrays (Lillestol et al., 2006) and beyond the CRISPR mini-arrays, to any means of intervirus competition. Several recent studies suggest that viruses indeed employ multiple, diverse mechanisms in the arms races with their competitors (Berngruber et al., 2010; Gentile et al., 2019; Mavrich and Hatfull, 2019; Montgomery et al., 2019), and investigation of these mechanisms using the game theoretical framework can enhance our understanding of the evolution of inter-virus conflicts.

## Data Availability Statement

All datasets for this study are included in the article/supplementary material.

## Author Contributions

JI and GF conceived the study. JI constructed the model. JI, GF, YW, and EK analyzed the results. JI and EK wrote the manuscript that was edited and approved by all authors.

## Conflict of Interest

The authors declare that the research was conducted in the absence of any commercial or financial relationships that could be construed as a potential conflict of interest.
